# Impact of Various High Fat Diets on Gene Expression and the Microbiome Across the Mouse Intestines

**DOI:** 10.21203/rs.3.rs-3401763/v1

**Published:** 2023-10-09

**Authors:** Jose Martinez-Lomeli, Poonamjot Deol, Jonathan R Deans, Tao Jiang, Paul Ruegger, James Borneman, Frances M. Sladek

**Affiliations:** University of California, Riverside

## Abstract

High fat diets (HFDs) have been linked to several diseases including obesity, diabetes, fatty liver, inflammatory bowel disease (IBD) and colon cancer. In this study, we examined the impact on intestinal gene expression of three isocaloric HFDs that differed only in their fatty acid composition – coconut oil (saturated fats), conventional soybean oil (polyunsaturated fats) and a genetically modified soybean oil (monounsaturated fats). Four functionally distinct segments of the mouse intestinal tract were analyzed using RNA-seq – duodenum, jejunum, terminal ileum and proximal colon. We found considerable dysregulation of genes in multiple tissues with the different diets, including those encoding nuclear receptors and genes involved in xenobiotic and drug metabolism, epithelial barrier function, IBD and colon cancer as well as genes associated with the microbiome and COVID-19. Network analysis shows that genes involved in metabolism tend to be upregulated by the HFDs while genes related to the immune system are downregulated; neurotransmitter signaling was also dysregulated by the HFDs. Genomic sequencing also revealed a microbiome altered by the HFDs. This study highlights the potential impact of different HFDs on gut health with implications for the organism as a whole and will serve as a reference for gene expression along the length of the intestines.

## Introduction

Over the last century, the dietary pattern in the U.S. has gradually shifted from a healthy diet to one with increased fat and decreased fiber. Along with an increase in the amount of fat being ingested, there has also been a change in the type of fat being consumed by Americans, with seed oils, high in polyunsaturated fatty acids (PUFAs), becoming the predominant source of dietary fat. In fact, the component of the U.S. diet that has increased the most over the last century is soybean oil ^1^. The major fatty acid component of soybean oil is the PUFA linoleic acid (LA, C18:2, omega-6). Our lab and many others have shown that high-fat diets (HFDs) can be linked to several diseases, such as obesity, diabetes, insulin resistance, fatty liver and susceptibility to inflammatory bowel disease (IBD) in both mice and humans ^2–5^. There are also many studies describing the impact of HFDs on the gut microbiota ^6,7^, physiological changes in the small intestine ^8^, intestinal permeability and gastrointestinal diseases ^9^. However, most gene expression studies analyze only one portion of the intestines or one type of HFD at a time ^10–12^.

Here, we used RNA-seq to examine the impact of three HFDs on gene expression in four functionally distinct segments of the mouse intestinal tract: the duodenum, jejunum, terminal ileum and proximal colon. The duodenum is responsible for breaking down the stomach acid and food mixture, while the jejunum absorbs sugars, amino acids, and fatty acids. The terminal ileum absorbs remaining nutrients, such as vitamin B12 and bile acids, and the proximal colon is the primary site for absorption of water and salts and microbial production of short chain fatty acids (SCFAs). All four parts of the intestine are also involved in xenobiotic and drug metabolism ^13^.

The HFDs used in this study are comparable to the current American diet in that they consist of 40% of calories from fat and are low in fiber. The first diet was formulated with coconut oil (saturated fat), the second with soybean oil (polyunsaturated fat, PUFA) and the third with a genetically modified soybean oil with a fatty acid composition similar to olive oil (monounsaturated fat). Each diet was compared to a low fat (13 kcal% fat), high-fiber vivarium chow as well as to each other. RNA-seq analysis revealed dysregulation of several nuclear receptor genes and other transcriptional regulators as well as xenobiotic/drug metabolism genes throughout the small and large intestines. There was also significant dysregulation of genes involved in epithelial barrier function, IBD and colon cancer. Network analysis showed an upregulation in metabolism genes and, interestingly, a downregulation in numerous genes involved in the immune system, particularly those related to bacterial and viral infections, including SARS-CoV-2, the pathogen responsible for the global COVID-19 pandemic. The expression of several genes related to signaling by neurotransmitters and the microbiome was dysregulated, and genome sequencing revealed alterations in the gut bacteria by the HFDs. Taken together, our results reveal a significant impact of dietary fat on the intestinal transcriptome and microbiome which could potentially contribute to disease as well as brain health.

## Materials and Methods

### Animals

Care and treatment of animals was in accordance with guidelines from and approved by the University of California, Riverside Institutional Animal Care and Use Committee (AUP #20140014). All the methods reported are in accordance with ARRIVE guidelines. The animals were treated as previously described ^3^. Briefly, male C57BL/6N mice were weaned at three weeks of age and assigned randomly to one of the four diets for 24 weeks – low fat Vivarium (VIV) chow; 40% kcal fat coconut oil (CO, 36 kcal% from coconut oil and 4 kcal% from soybean oil to provide the essential fatty acids LA and ALA), 40% kcal CO plus soybean oil (SO + CO, 21 kcal% fat calories from coconut oil and 19 kcal% from soybean oil, resulting in 10 kcal% from LA, comparable to the amount of LA in the current American diet ^14^); 40% kcal CO plus genetically modified soybean oil (Plenish) which has low LA (PL + CO, conventional soybean oil was replaced on a per gram basis with the genetically modified (GM) High Oleic Soybean Oil Plenish (DuPont Pioneer, Johnston, IA). See Supplementary Table S1 for a comparison of the diets and Deol et al., 2017 ^3^ for the complete composition of the diets. Metabolic parameters of the mice, including body weight, glucose tolerance, insulin resistance and fatty liver were reported previously ^3^. At the end of the study, animals were euthanized by CO_2_ inhalation followed by cervical dislocation. Intestinal tissue was excised immediately and put in RNALater for 24 hours at room temperature and then stored at −80°C.

### RNA-seq

The tissues for RNA sequencing (RNA-seq) were duodenum (DUO, 1 cm immediately downstream of the gastroduodenal junction), jejunum (JEJ, 1 cm at the approximate middle of the remainder of the small intestine), terminal ileum (TI, 1 cm immediately upstream of the ileo-cecal junction), and proximal colon (PC, 1 cm immediately downstream of the ileo-cecal junction). RNA extraction from each of the four portions of the intestines for three VIV chow-fed mice and four mice on each of the three HFDs (60 total samples) was carried out as previously described ^2^. Total RNA was isolated from samples of each tissue (DUO, JEJ, TI and PC) using a miRNeasy kit (Qiagen, Inc., Valencia, CA) and evaluated for purity and concentration by NanoDrop (Wilmington, DE) and Agilent 2100 Bioanalyzer (Santa Clara, CA). Poly(A) + RNA (4 μg) with an RNA Integrity Number (RIN) of 7.8 or higher was used to construct sequencing libraries with the TruSeq Long RNA Sample Prep Kit (Illumina, San Diego, CA). RNA libraries were validated for RNA integrity by Bioanalyzer, pooled in equimolar amounts, and sequenced on an Illumina HiSeq 2000 at the UCR Genomics Core to generate 50 bp paired-end reads. Three biological replicates were sequenced for the Vivarium Chow diet (VIV) and four for all the HFDs (CO, SO + CO, and PL + CO). On average ~ 16 million reads were acquired for each biological replicate. The raw data are publicly available in Gene Expression Omnibus (GEO), accession number GSE220302.

### Differential gene expression analysis of RNA-seq data

Reads were aligned to the mouse reference genome (mm10) with STAR v2.5.0a using default parameters ^15^. Raw read counts were calculated with STAR using the GeneCounts option of the quantMode parameter since the libraries were unstranded. Library normalization was performed with EDASeq ^16^; within-lane normalization on GC content was performed with the LOESS method and between-lane normalization was performed with the non-linear full quantile method. Normalization factors from EDASeq were used for differential expression analysis with DESeq2 ^17^. Normalized read counts, FPKM (fragments per kilobase per million), and r-log (regularized log transformation) results were generated for downstream analysis.

The list of genes used in the heatmaps for nuclear receptors, epithelial barrier, IBD, colon cancer, microbiome and COVID-19 were obtained from the NCBI website (Supplementary Table S6). Differentially expressed genes (DEGs) between any two diets (p-adj ≤ 0.05) were identified in the RNA-seq data and displayed in the respective heatmaps, generated using the Pheatmap package in R ^18^ and row-normalized before plotting, unless noted otherwise. Python library “Plotly” was used to generate scatter plots for each individual gene ^19^. PCA analysis, bar plots and Venn diagrams were created using the Python library ‘matplotlib’. Volcano plots were generated using the ggplot2 package from R ^20^. Colored spots are DEGs with (p-adj ≤ 0.05 & abs(Log2FC) ≥ 0.05). Genes symbols for outliers are highlighted and have a value in the top 95% in −Log10(p-adj) and abs(Log2FC) ≥ 1.5 in the given comparison. StringApp ^21^ from Cytoscape (Version 3.8.2) ^22^ was used to analyze and visualize potential interactions between DEGs among the different diets and tissues in the KEGG ^23^ and Reactome pathways ^24^ (FDR ≤ 0.05); a medium interaction score of 0.4 (out of 0 to 1) in the StringApp was required. Mouse Genome Informatics (MGI) and GeneCards: The Human Gene Database were used to identify the full name of a gene, as well as its functions and associated diseases ^25,26^.

### Microbiome analysis

The bacterial collection protocol, DNA extraction and bacterial rRNA internal transcribed spacer (ITS) analysis was performed as previously described ^27^ except that bacteria were collected from the small intestine or colon of male mice fed the different diets (VIV, SO, SO + CO, PL, PL + CO) for 24 weeks – the same ones used for the RNA-seq. Only the top 12 genus-level of operational taxonomic units (OTU) were plotted as mean percentage compositions for each treatment group; the remaining OTUs were combined under “Other”. DNA sequencing data of the microbiome is publicly available at SRA BioProject, Accession #PRJNA615924.

## Results

**Figure 1:**
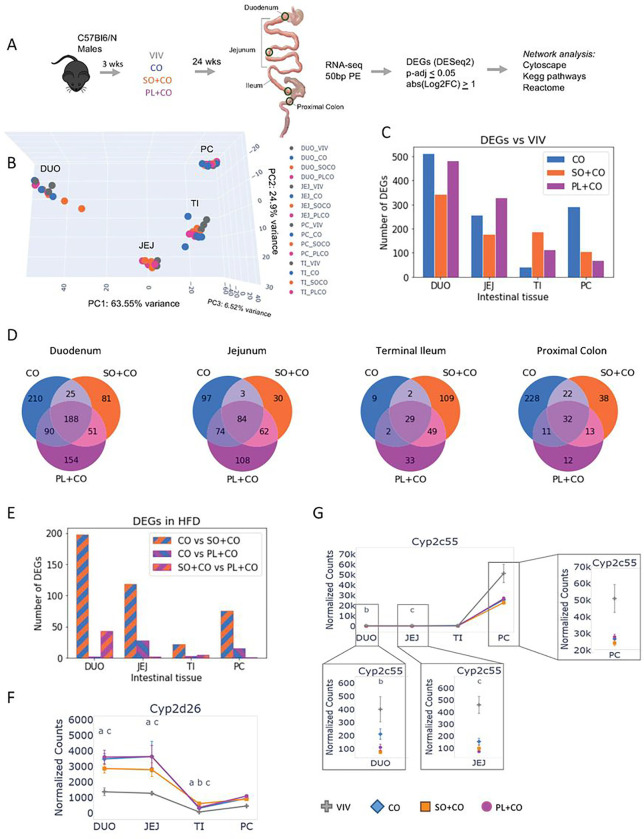
Differential impact of HFDs on gene expression across different parts of the intestines **A.** Work-flow: Male C57Bl6N male mice were weaned at 3 weeks of age to either a regular chow diet (VIV) or one of the three high fat diets – CO, coconut oil; SO+CO, soybean oil enriched; PL+CO, low-LA soybean oil (Plenish) enriched. One centimeter of each tissue was used to perform RNA-seq (regions indicated with a circle). Post sequencing analysis was done as indicated. N=3 per tissue for VIV and 4 per tissue for the HFDs. See Supplementary Table S1 for diet composition. **B.** 3D principal component analysis (PCA) showing differential effects of the diets on different parts of the intestines. **C.** Bar plot showing the number of differentially expressed genes (DEGs, up and down regulated) (p-adj ≤0.05 and absolute fold change ≥2 (abs(Log2FC) ≥ 1)) in three HFD vs VIV chow in different parts of the intestines. See Supplementary Tables S2-S5 for complete comparison of genes between diets and Supplementary Figure S2 for volcano plots of the most dysregulated genes. **D.** Venn diagrams showing the overlap of the DEGs (p-adj <0.05, Log2FC>1) in the indicated diet comparisons across the tissues. See Supplementary Figure S1 for Venn analysis between HFDs. **E.** Bar plot showing the number of differentially expressed genes (DEGs, up and down regulated) (p-adj ≤0.05 and absolute fold change ≥2 (abs(Log2FC) ≥ 1) between the three HFD comparisons in different parts of the intestines. **F-G**. Line graph of the average normalized read counts with standard deviation (SD) of *Cyp2d26* (**F)** and *Cyp2c55* (**G)** in various parts of the intestines on the indicated diets (VIV, CO, SO+CO, PL+CO). Significantly different levels of expression between the diets within a given tissue denoted by p-adj ≤ 0.05 and are indicated as follows: a (VIV vs Co); b (VIV vs SO+CO); c (VIV vs PL+CO); d (CO vs SO+CO); e (CO vs PL+CO); f (SO+CO vs PL+CO). See Supplementary Figures S3 and S4 for heatmaps and line graphs of additional *Cyp, Gst* and *UGT* genes.

Male C57BL6/N mice were fed one of four diets for a period of 24 weeks and gene expression was examined in different portions of the intestines (Fig. 1A). The diets included a low-fat Vivarium chow (VIV) and three high-fat diets (HFDs) with 40% of calories derived from fat: a coconut oil diet (CO) composed of primarily saturated fats, specifically lauric acid (C14:0) and myristic acid (C12:0), with a small amount of soybean oil to provide the essential fatty acid linoleic acid (LA) (2% kcal); a soybean oil-enriched diet (SO + CO) with a high LA content (10% kcal); and a diet enriched in a genetically modified soybean oil (PL + CO) known as Plenish, which has a low LA (1.4% kcal) and high oleic acid content (~ 14% kcal) ^28^ (Supplementary Table S1). Previous analysis of these mice revealed that the soybean oil diet (SO + CO), and to a lesser extent the Plenish diet (PL + CO), induced obesity, diabetes, insulin resistance, and fatty liver, while the isocaloric CO diet had minimal adverse metabolic effects despite similar caloric intake as the other HFDs ^3^. RNA-seq was performed on a segment of each of the four tissues: duodenum (DUO), jejunum (JEJ), terminal ileum (TI), and proximal colon (PC). Differentially expressed genes (DEGs) were identified using DeSeq2, with statistical significance determined by a p-adjusted value of less than 0.05 and an absolute fold change greater than 2 (p-adj < 0.05 & Log2FC > 1.0). The DEGs (p-adj < 0.05) were further analyzed using network analysis in Cytoscape, incorporating the KEGG and Reactome databases (Fig. 1A).

### HFDs alter gene expression in a differential fashion across the intestinal tract, including drug metabolism genes

Principal Components Analysis (PCA) of the 60 RNA-seq datasets revealed that the transcriptomes were primarily grouped based on tissue, with smaller variations observed between the dietary groups (Fig. 1B). Nonetheless, a considerable number of DEGs were identified when any of the three HFDs were compared to the VIV chow within a specific tissue (Fig. 1C). The duodenum (DUO) exhibited the greatest number of DEGs in all three HFD vs. VIV chow comparisons (CO: 513; SO + CO: 345; PL + CO: 483). The jejunum (JEJ) also had a substantial number of DEGs, albeit fewer than the duodenum (CO: 258; SO + CO: 179; PL + CO: 328), while the terminal ileum (TI) had a lower number of DEGs, except for the SO + CO vs. VIV chow (CO: 42; SO + CO: 189; PL + CO: 113). In contrast, the proximal colon (PC) displayed the largest number of DEGs in the CO vs. VIV comparison (CO: 293; SO + CO: 105; PL + CO: 68) (Fig. 1C). A Venn analysis revealed a moderate to minimal overlap in DEGs between the different HFDs and the VIV chow, ranging from 188 genes in the duodenum to 29 genes in the terminal ileum (Fig. 1D). These findings indicate that diets composed of different fats have distinct impacts on specific segments of the intestines.

Comparison between each of the three HFDs showed that CO vs. SO + CO consistently yielded the greatest number of DEGs (DUO: 198; JEJ: 118; TI: 22; PC: 75) (Fig. 1E). In contrast, CO vs. PL + CO exhibited a surprisingly low number of DEGs (ranging from 2 to 28) in all four tissues, except for the duodenum, which had 43 DEGs. Venn analysis of the pairwise comparisons between the HFDs revealed no overlap in DEGs among all three comparisons and relatively limited overlap between any two comparisons (Supplementary Figure S1).

Volcano plot analysis identified individual genes with significant fold change in various HFD vs. VIV chow comparisons, including several cytochrome P450 (*Cyp*) genes (Supplementary Figure S2). For example, *Cyp2d26* was expressed at higher levels in the small intestines than the proximal colon and significantly upregulated by all three HFDs (Fig. 1F). In contrast, *Cyp2c55* was expressed at much higher levels in the proximal colon than the small intestines and the HFDs tended to decrease expression, although it did not reach significance (Fig. 1G). Several other *Cyp* genes (*Cyp4a10, Cyp4a31, Cyp4a32, Cyp4f15, Cyp2j6, Cyp2j9*) were upregulated primarily in the duodenum by all three HFDs while a few genes were dysregulated in the jejunum by one or more HFDs (*Cyp2u1, Cyp2c29, Cyp4f16*) (Supplementary Figure S3). Expression of other *Cyp* genes as well as Phase 2 *Ugt* and *Gst* genes also varied across the intestines on the VIV chow and in response to the different HFDs, with a very modest impact on relatively few Phase 2 genes (e.g., *Gstm1, Gsta4, Ugt1a9, Ugt1a7, Ugt2b36*) and a greater impact on a number of *Cyp* genes (Supplementary Figure S4).

### Differential expression of nuclear receptors across the intestinal tract and in response to HFD

**Figure 2:**
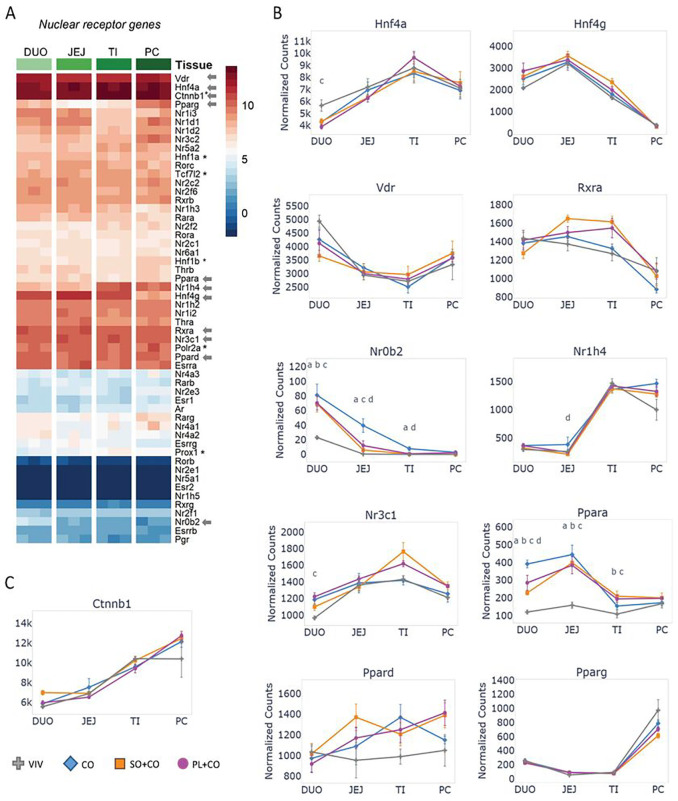
Differential expression of nuclear receptors across the intestinal tract and in different HFDs **A.** Non row-normalized heatmap showing levels of all 48 nuclear receptors (NR) across the tissues in mice fed VIV chow, sorted by levels in the duodenum (DUO) and compared to non NR transcription factors (*). Normalized read counts across three biological replicates are shown. JEJ, Jejunum; TI, Terminal Ileum; PC, Proximal colon. Arrows, genes plotted in figure. Arbitrary scale of relative expression is shown. See Supplementary Figure S5 for additional heatmaps of nuclear receptors. **B.** Line graphs showing normalized read counts with standard deviation (SD) of select NRs in various parts of the intestines on the indicated diets. Significantly different genes between diets within a given tissue (p-adj ≤ 0.05) are indicated as follows: a (VIV vs Co); b (VIV vs SO+CO); c (VIV vs PL+CO); d (CO vs SO+CO); e (CO vs PL+CO); f (SO+CO vs PL+CO). **C.** As in B but for beta-catenin (*Ctnnb1*).

Several members of the nuclear receptor (NRs) superfamily of ligand-dependent transcription factors are known to regulate CYP genes and play important roles in the development and function of the intestinal tract, as well as pathologies such as IBD and colon cancer ^29,30^. To determine their relative expression in different parts of the intestines we compared all 48 NRs across the four intestinal tissues in the mice fed VIV chow in a non row-normalized heatmap and included several non-NR transcription factors (TFs) known to play a role in intestinal physiology (*Ctnnb1, Hnf1a, Hnf1b, Polr2a, Prox1, Tcf7l2*). The most highly expressed NR gene throughout the intestines is hepatocyte nuclear receptor 4 alpha (*Hnf4a*) – its expression was greater than that of RNA polymerase 2 (*Polr2a*) and nearly as high as beta-catenin (*Ctnnb1*) – followed by the vitamin D3 receptor (*Vdr*), *Hnf4g*, and *Rxra* (Fig. 2A). This relative order was maintained across the three HFDs as well (Supplementary Figure S5). Some NR genes (e.g., *Hnf4a, Nr1h4, Pparg*) are expressed at lower levels in duodenum or jejunum, and at higher levels further along the intestinal tract while others (e.g., *Hnf4g, Vdr, Nr0b2, and Ppara*) have a relatively high level of expression in the beginning of the intestines and then decrease in the latter portions (Fig. 2BC). Others, such as *Rxra*, which is a heterodimeric partner for many other NRs, have a fairly consistent level of expression across the four tissues, decreasing only in the proximal colon (Fig. 2B).

Among the top four most highly expressed NRs, the only one that showed differential expression among the different diets was *Hnf4a*. Its expression in the duodenum was decreased in the intestines of mice fed any of the three HFDs compared to VIV chow (Fig. 2B). *Nr0b2* (short heterodimeric partner, SHP) which acts as a transcriptional repressor, the bile acid receptor (FXR, *Nr1h4*) and the glucocorticoid receptor (GR, *Nr3c1*), which plays a critical role in the stress response, all showed a significant difference from VIV chow in one or more HFD in at least one portion of the intestines (Fig. 2B). In contrast, there was no significant difference in *Ctnnb1* expression among the various diets, which is noteworthy as both HFD and mutations in the Wnt-Beta-catenin pathway are risk factors for colon cancer in humans (Fig. 2C) ^31^.

Finally, we examined the PPARs, which are known to play a role in the regulation of nutrient transport from the lumen into the body and have fatty acids as their ligands. While *Ppard* and *Pparg* did not show any significant difference in expression between diets within a given tissue, *Ppara* expression was significantly increased in the duodenum and jejunum in all three HFDs. It was also increased in CO vs PL + CO in the duodenum and in SO + CO or PL + CO vs VIV chow in the terminal ileum (Fig. 2B).

### HFD impacts the expression of intestinal epithelial barrier function genes

**Figure 3:**
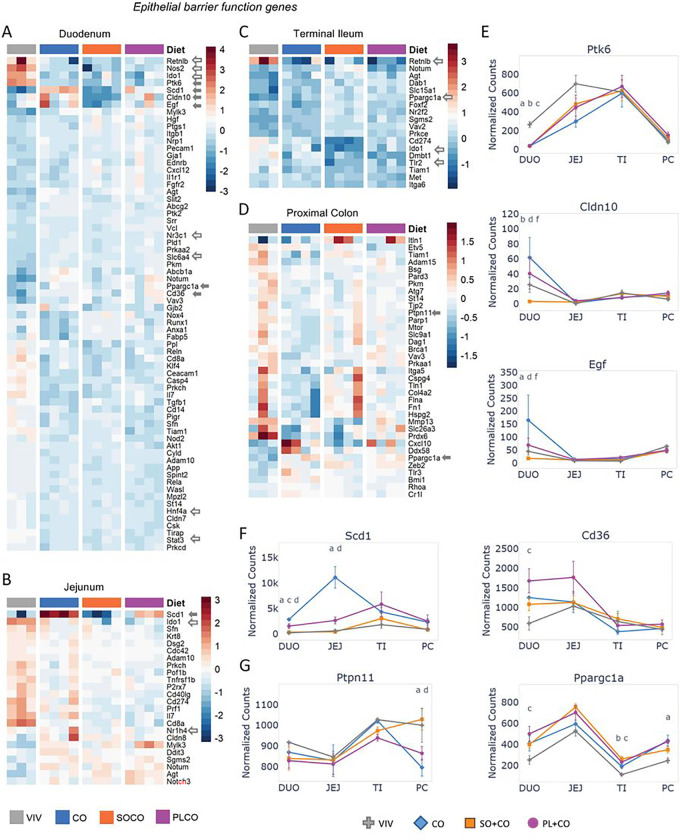
HFDs impact the expression of epithelial barrier function genes across the intestines **A – D.** Heatmaps of genes involved in epithelial barrier function in the indicated portions of the intestines of mice fed either low fat VIV chow or one of the three HFDs. Included are genes that are significantly different between any two diets (p-adj ≤ 0.05). Solid arrow, plotted in figure; open arrow, plotted in a subsequent figure. Arbitrary scale of relative expression is shown. See Supplementary Table S6 for a complete list of genes. **E-G.** Line graphs showing normalized read counts with standard deviation (SD) of select genes on the indicated diets. Genes with significantly different levels of expression between the diets within a given tissue (p-adj ≤ 0.05) are indicated as follows: a (VIV vs CO); b (VIV vs SO+CO); c (VIV vs PL+CO); d (CO vs SO+CO); e (CO vs PL+CO); f (SO+CO vs PL+CO).

Formation and maintenance of a healthy epithelial barrier is an important physiological function of the intestines. To analyze the effect of diet on intestinal barrier function, we used a list of 444 genes from NCBI (Supplementary Table S6) and identified 123 genes that are significantly dysregulated (p-adj < 0.05) between any two dietary groups (Fig. 3A–D). The duodenum had the greatest number of dysregulated genes (mostly downregulated) across the different diets (68 genes). Several genes exhibited lower levels of expression in one or more HFDs compared to the VIV chow in the duodenum – e.g., *Ptk6* (Protein tyrosine kinase 6), *Cldn10* (Claudin 10), *Egf* (epidermal growth factor). In contrast, *Cd36* (cluster of differentiation 36, a long chain fatty acid transporter) showed increased expression in PL + CO vs VIV chow in the duodenum while NR co-activator *Ppargc1a* (PPARG Coactivator 1 Alpha) showed elevated expression in one of more HFD in all parts of the intestines except the jejunum (Fig. 3E). Considering that PGC1A is a co-activator of HNF4A and the PPARs ^32^, these diet-induced changes in *Ppargc1* expression could amplify the effects of the HFDs on the NRs.

The jejunum, responsible for lipid digestion and absorption in the intestines, displayed a pattern where most of the 24 DEGs were between VIV chow and the three HFDs, with little difference between the HFDs (Fig. 3B). The exception was *Scd1*, which had much higher expression in the CO diet compared to the other HFDs and the VIV chow (Fig. 3F), consistent with the function of SCD1, a desaturase enzyme that introduces double bonds into saturated fatty acids.

The terminal ileum has the least HFD-dysregulated genes (18 DEGs) related to barrier function (Fig. 3C). The most dysregulated gene was Resistin-like molecule (RELM) β (*Retnlb*), a cysteine-rich cytokine that plays a role in insulin resistance, gastrointestinal nematode resistance, barrier integrity and susceptibility to inflammation ^33^. *Retnlb* expression was decreased by all three HFDs in the terminal ileum (as well as the duodenum) (see Fig. 6B). Since the terminal ileum is the region of the intestines that harbors many bacteria, viruses, and other pathogens, a downregulation in *Retnlb* caused by a HFD could weaken the body’s defenses. Another gene showing differential expression with HFDs in the proximal colon is the IBD susceptibility gene *Ptpn11* (down in CO vs VIV and SO + CO), which encodes a tyrosine phosphatase involved in the homeostasis of epithelial barrier cells ^34^ (Fig. 3G).

### HFD impacts the expression of genes associated with IBD and colon cancer

**Figure 4:**
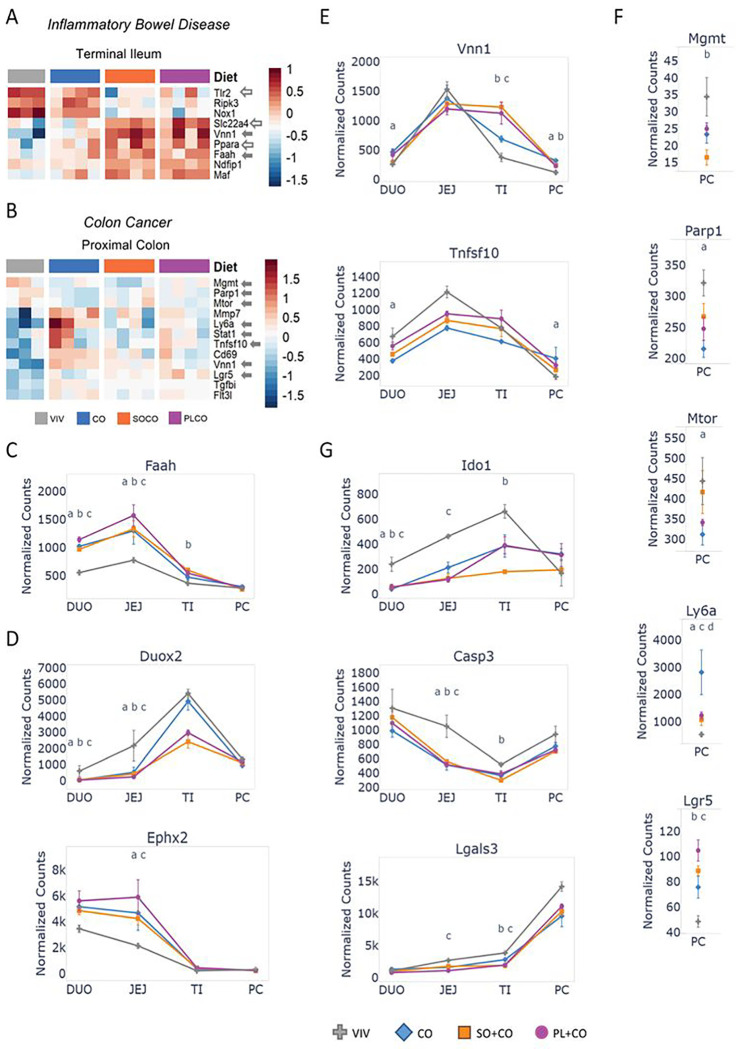
HFDs alter the expression of genes associated with Inflammatory Bowel Disease (IBD) and colon cancer **A-B.** Heatmaps of genes involved in IBD and colon cancer in the terminal ileum and proximal colon, respectively, of mice fed either low fat VIV chow or one of the three HFDs. Included are genes that are significantly different between any two diets (p-adj ≤0.05). N= 3 for Viv and 4 for HFDs per tissue. Solid arrows, plotted in this figure; open arrows, plotted in a subsequent figure. Arbitrary scale of relative expression is shown. See Supplementary Table S6 for a complete list of genes and Supplementary Figure S6 for additional heatmaps of IBD and colon cancer genes. **C-G.** Line graphs showing normalized read counts with standard deviation (SD) of select genes in various parts of the intestines (only proximal colon is shown in **F**) on the indicated diets for IBD and colon cancer. Genes with significantly different levels of expression between the diets within a given tissue (p-adj ≤ 0.05) are indicated as follows: a (VIV vs Co); b (VIV vs SO+CO); c (VIV vs PL+CO); d (CO vs SO+CO); e (CO vs PL+CO); f (SO+CO vs PL+CO).

The expression of genes involved in IBD (141 genes) and colon cancer (192 genes) was also impacted by the HFDs (Fig. 4AB and Supplementary Figure S6). Interestingly, in terms of IBD-related genes, the terminal ileum was impacted the most by the HFDs, consistent with this portion of the gut being frequently inflamed in Crohn’s Disease, a form of IBD (Fig. 4A). *Tlr2* (Toll-like receptor 2), *Ripk3* (receptor interacting serine/threonine kinase 3), and *Nox1* (NADPH oxidase 1) all decreased expression in the SO + CO and PL + CO diets compared to the VIV chow and CO diet. In contrast, *Slc22a4* (a member of the solute carrier family), *Vnn1* (vanin 1), *Faah* (fatty acid amide hydrolase), *Ndfip1* (Nedd4 Family Interacting Protein 2), *Maf* (bZIP transcription factor) showed increased expression in the two soybean oil diets (Fig. 4A,C,E). Noteworthy IBD-related genes in the duodenum and/or jejunum that were affected by the HFDs include *Duox2* (dual oxidase 2), a member of the NADPH oxidase family which was downregulated by the HFDs, and *Ephx2* (epoxide hydrolase 2), which converts fatty acid epoxides to bioactive dihydrodiols, was upregulated by the HFDs (Fig. 4D).

The HFDs also affected the expression of cancer-related genes in the proximal colon (and other parts of the intestines) including *Vnn1* (vanin 1), a pantetheinase with roles in oxidative stress and inflammation ^35^, and *Tnfsf10* (tumor necrosis factor ligand superfamily, member 10) (Fig. 4E). Genes specific to colon cancer and altered only in the proximal colon include DNA repair enzymes *Mgmt* (O-6-Methylguanine-DNA Methyltransferase) and *Parp1* (Poly(ADP-Ribose) Polymerase 1), *Mtor*, a mediator of response to cellular stress including DNA damage – all were downregulated by one or more HFDs. In contrast, *Ly6a* (*L*ymphocyte Antigen 6A), which regulates T cell proliferation, and *Lgr5*, a prominent marker for mitotically active crypt intestinal stem cells involved in the Wnt signaling pathway, were upregulated (Fig. 4F). Finally, there were several genes related to colon cancer that were altered by the HFDs but only in the small intestines. For example, *Ido1* (indoleamine 2,3-dioxygenase 1) is the first and rate-limiting step in tryptophan catabolism and plays a role in antimicrobial and anti-tumor defense, neuropathology and immunoregulation, *Casp3* (caspase 3) is a key executor of apoptosis and *Lgals3* (galectin 3) plays a role in innate immunity and T-cell regulation and exhibits antimicrobial activity against bacteria and fungi. All three were downregulated by the HFDs (Fig. 4G).

### Network analysis reveals an impact of HFDs on the immune system as well as metabolism

**Figure 5:**
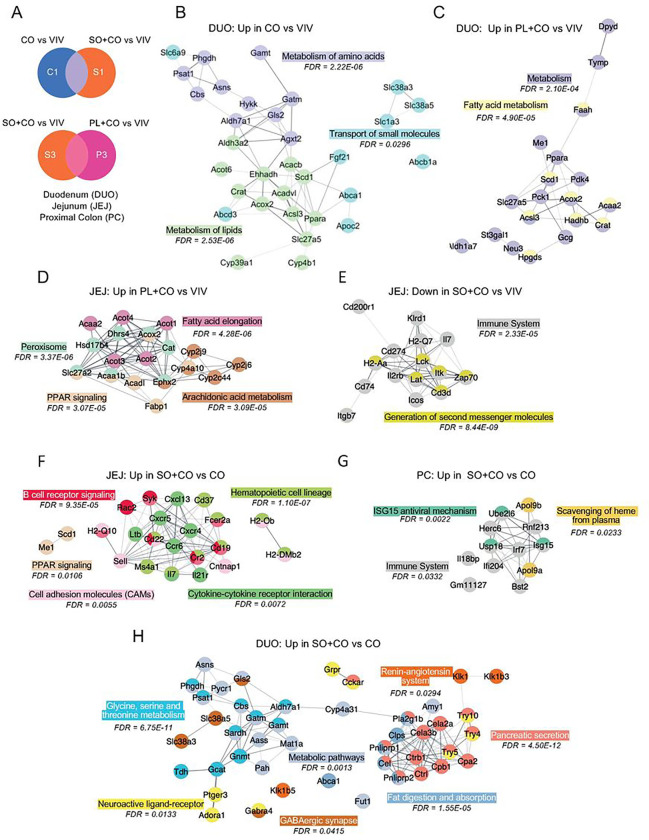
Network analysis of differentially expressed genes (DEGs) in various HFDs and vivarium chow in various parts of the intestines **A.** Venn diagram of pairwise comparisons of differentially expressed genes (DEGs) (p-adj ≤ 0.05) between each HFD (CO, SO+CO, PL+CO) and the low-fat Vivarium chow (VIV). **B-H.** Networks of DEGs either up or down-regulated in the various tissues in the indicated portions of the Venn diagram in (**A**). C1: dysregulated in CO vs. VIV but not in SO+CO vs. VIV; S1: dysregulated in SO+CO vs. VIV but not in CO vs. VIV; C2: dysregulated in CO vs. VIV but not in PL+CO vs. VIV; P2 dysregulated in PL+CO vs. VIV but not in CO vs. VIV; S3 dysregulated in SO+CO vs. VIV but not in PL+CO vs. VIV; P3 dysregulated in PL+CO vs. VIV but not in SO+CO vs. VIV. Networks were identified in Cytoscape: Reactome (**B**, **C**, **D**, **E**, **G**) or KEGG (**F**, **H**). Individual FDRs for the indicated pathways are shown. See Supplementary Figure S7 for additional networks.

To obtain a more detailed understanding of the pathways impacted by HFDs in the different parts of the intestines, we conducted a Venn analysis of the DEGs in different diet comparisons in each tissue followed by Stringapp in Cytoscape to identify networks of genes, utilizing either the Reactome or the KEGG pathway databases (Fig. 5, Supplementary Figure S7). In the duodenum, genes upregulated in the CO vs. VIV comparison but not in SO + CO (C1) were involved in the metabolism of amino acids and lipids, as well in the transport of small molecule pathways (Fig. 5B). Additional metabolic categories, especially involving fatty acids, were identified in the PL + CO vs VIV comparison (Fig. 5C). In contrast, downregulated genes in the duodenum in the CO vs VIV comparison were associated with T cell receptor (TCR) signaling and the innate immune system, while genes down in the SO + CO vs VIV comparison were found in pathways related to pancreatic secretion, chemical carcinogenesis, linoleic acid metabolism and fat digestion and absorption (Supplementary Figure S7BC). Similarly, in the jejunum, there were many upregulated genes in HFD vs VIV, including fatty acid elongation, arachidonic acid metabolism and PPAR signaling and peroxisome (Fig. 5D) and fatty acid metabolism and Phase I genes (Supplementary Figure S7E). In contrast, as in the duodenum, the down regulated genes in the jejunum were related to the immune system, second messenger molecules, cytokine signaling and herpes simplex infection (Fig. 5E, Supplementary Figure S7FG). In contrast, the SO + CO vs CO comparison in the jejunum revealed upregulated genes associated with the immune system (B cell receptor signaling, hematopoietic cell lineage, cytokine-cytokine receptor interactions) as well as PPAR signaling and cell adhesion molecules (Fig. 5F). The same comparison in the proximal colon (SO + CO vs CO) also showed upregulated genes related to the immune system, ISG15 antiviral mechanism and scavenging of heme from plasma (Fig. 5G). In the SO + CO vs CO comparison, the duodenum yielded a completely different mix of upregulated metabolic pathways (including glycine, serine and threonine metabolism), fat digestion and absorption, pancreatic secretion, the renin-angiotensin system (RAS) and, intriguingly, GABAergic synapse and neuroactive ligand-receptor (Fig. 5H) as well as oxidative phosphorylation (Supplementary Figure S7D). Lastly, there was a network of genes up in the proximal colon in the SO + CO vs CO comparison involved in herpes simplex infection, RIG-I-like receptor signaling and cytosolic DNA sensing (Supplementary Figure S7H). There were no significant networks among the genes in the terminal ileum.

### Impact of HFDs on the gut microbiome

**Figure 6:**
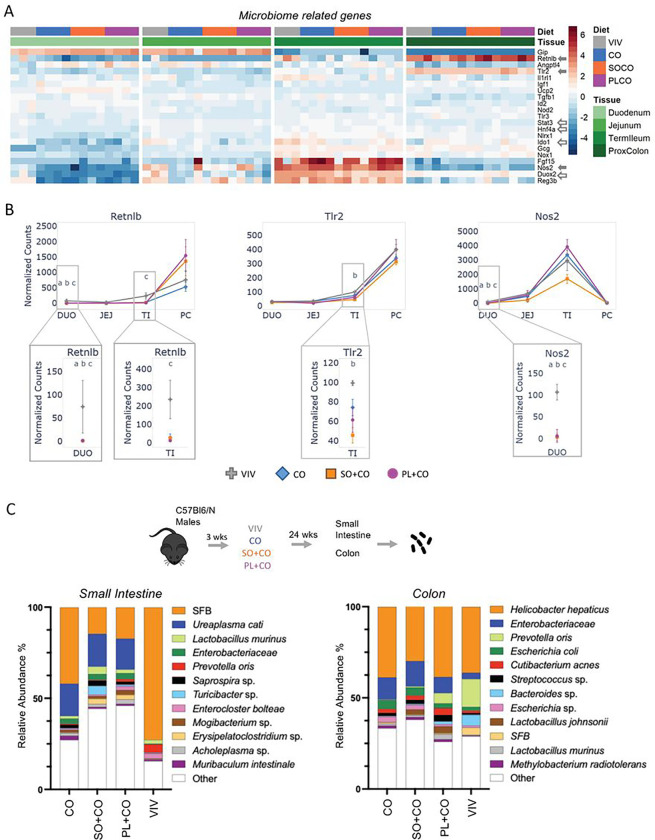
Impact of HFDs on the gut microbiome and related host genes **A.** Heatmap of significantly dysregulated genes involved in the microbiome response in the host. Included are genes that are significantly different between any two diets (p-adj ≤ 0.05). Solid arrows, plotted in this figure; open arrows, plotted in a subsequent figure. Arbitrary scale of relative expression is shown. See Supplementary Table S6 for a complete list of genes. **B.** Line graphs showing normalized read counts with standard deviation (SD) of select genes in various parts of the intestines on the indicated diets. Genes with significantly different levels of expression between the diets within a given tissue (p-adj ≤ 0.05) are indicated as follows:(VIV, CO, SO+CO, PL+CO). a (VIV vs Co); b (VIV vs SO+CO); c (VIV vs PL+CO); d (CO vs SO+CO); e (CO vs PL+CO); f (SO+CO vs PL+CO). **C.** Taxa plots showing differentially abundant bacteria from host-associated intestinal epithelial cells in the small intestine or colon of mice fed the different diets (CO, SO+CO, PL+CO, VIV). Values in taxa plots are % IlluminaITS rRNA gene reads from intestinal epithelial cells from the indicated tissue. n = 11–12 mice for each of the four diets.

Since HFDs are known to impact the microbiome ^36^, we generated a heatmap of microbiome-related genes that showed differential expression between any two diets (Fig. 6A). For example, *Retnlb* (resistin-like beta), which has antimicrobial properties, showed consistently high expression in the proximal colon compared to other tissues; it also showed decreased expression by one or more HFD in the duodenum and terminal ileum (Fig. 6B). *Tlr2* (toll like receptor 2), a pattern recognition gene, and *Nos2* (nitric oxide synthase 2), which plays a role in immunity against bacteria, fungi and viruses, were also decreased in one or more HFD in the terminal ileum and duodenum, respectively (Fig. 6B).

Microbiome analysis of the small intestine and colon for the HFDs and VIV chow revealed the presence of many species of bacteria, with their relative abundance influenced by the diet (Fig. 6C). Importantly, there was an increase in populations of various pathogenic and opportunistically pathogenic bacteria in both the small intestines and the colon in the HFDs compared to VIV chow – *Ureaplasma cati, Turicibacter sp*. *and Erysipelatoclostridium sp*. in the small intestines and *Enterobacteriaceae* in the colon ^37–40^. There was also a notable decrease in bacteria with the HFDs that are typically considered to be beneficial (although their impact on host health is not fully understood yet) – Segmented filamentous bacteria (SFB) in the small intestines and *Prevotella oris* in the colon ^41,42^.

### Impact of HFDs on the expression of genes involved in COVID-19

**Figure 7:**
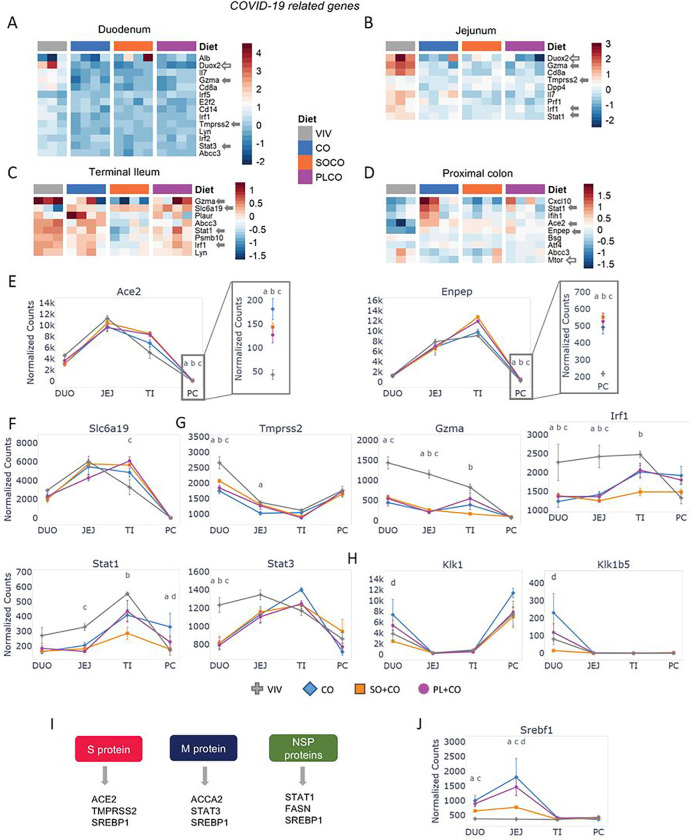
HFDs impact the expression of genes involved in SARS-CoV-2 across the intestinal tract **A-D.** Heatmaps of significantly dysregulated genes involved in COVID-19. Included are genes that are significantly different between any two diets (p-adj ≤0.05). Solid arrows, plotted in this figure; open arrows, plotted in other (main) figures. Arbitrary scale of relative expression is shown. See Supplementary Table S6 for a complete list of genes. **E-H.** Line graphs showing normalized read counts with standard deviation (SD) of select genes in various parts of the intestines on the indicated diets involved in COVID-19. Genes with significantly different levels of expression between the diets within a given tissue (p-adj ≤0.05) are indicated as follows: a (VIV vs Co); b (VIV vs SO+CO); c (VIV vs PL+CO); d (CO vs SO+CO); e (CO vs PL+CO); f (SO+CO vs PL+CO). **I.** Interaction between indicated host proteins and SARS-Co-V2 viral proteins. **J.** As in E-H but for *Srebf1*.

Although COVID-19 primarily affects the respiratory system, it can also impact the intestinal tract, leading to diarrhea, inflammation and septic shock ^43^. Furthermore, patients with COVID-19-related diarrhea are more likely to require hospitalization and experience a more severe infection ^43^. Heatmaps revealed several COVID-19-related genes that were dysregulated by one or more of the HFDs (Fig. 7A–D), including *Ace2* (angiotensin-converting enzyme 2) and *Enpep* (glutamyl aminopeptidase) (Fig. 7E). In the proximal colon, both genes exhibited a significant increase in expression in HFDs compared to VIV chow. *Slc6a19* (solute carrier family 6 member 19) showed increased expression in the terminal ileum in PL + CO vs. VIV chow (Fig. 7F). In contrast, *Tmprss2* (transmembrane Serine protease 2), *Gzma* (granzyme A), *Irf1* (interferon regulatory factor 1), *Stat1* and *Stat3* (signal transducer and activator of transcription 1/3) displayed decreased expression in one or more HFDs compared to VIV chow in various sections of the intestines (Fig. 7G). Moreover, two COVID-19-related genes, *Klk1* and *Klk1b5*, identified in the Renin-angiotensin system (RAS) in the network analysis (Fig. 5H), were upregulated by the CO diet in the duodenum (Fig. 7H). Kallikreins are serum serine proteases that play an important role in the vascular system and have been proposed as therapeutic targets for COVID-19 ^44,45^.

To further investigate the impact of HFDs on intestinal health during COVID-19, we utilized the BioGRID database ^46^ to identify interactions between host proteins/genes dysregulated by the HFDs and viral proteins of SARS-CoV-2, the causative agent of COVID-19. These interactions involved ACE2, TMPRSS2, SREBPF1 with the viral S protein; ACCA2, STAT3, and SREBPF1 with the viral M protein; and STAT1, FASN, and SREBPF1 with the viral NSP proteins (Fig. 7I). The expression of *Srebf1* (sterol regulatory element binding transcription factor 1), was significantly increased in the duodenum and jejunum in response to HFDs (Fig. 7J).

## Discussion

To our knowledge, this is the first comprehensive RNA-seq analysis conducted in four different sections of the intestines (duodenum, jejunum, terminal ileum and proximal colon) and comparing three distinct HFDs to a standard low-fat diet. The HFDs are unique in that they are not the standard diet made from excessive amounts of lard (50–60 kcal% fat) as is typically used in rodent studies. Rather, they are formulated with an amount of fat closer to that consumed by Americans (40 kcal%) ^47^ and using the most prevalent cooking oil used in the United States, soybean oil (SO) which is high in the polyunsaturated fat LA (C18:2), a genetically modified soybean oil Plenish (PL) low in LA and high in the monounsaturated fat oleic acid (C18:1) that is found in olive oil, and coconut oil (CO) consisting of saturated fat. Significant differences between diets within tissues were observed, with different diets impacting the expression of different genes in different parts of the intestines. Interestingly, the SO + CO diet resulted in a greater number of dysregulated genes compared to the CO diet in a variety of different pathways in different parts of the intestines than did the PL + CO diet, suggesting that excess LA has a greater impact than oleic acid (Figs. 1, 5), consistent with differential effects of SO and PL we have observed previously in terms of obesity, diabetes and colitis ^3,4^

**Figure 8:**
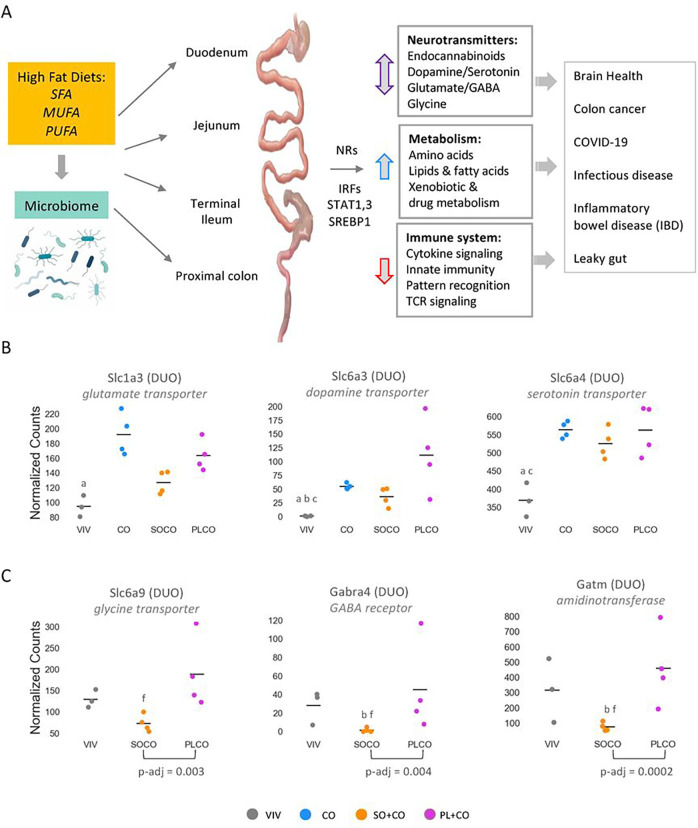
Overview of impact of HFDs on microbiome and gene expression. **A.** Overview of the role various HFDs may play in the development of disease by impacting the indicated pathways along the intestinal tract. SFA, saturated fatty acids; MUFA, monounsaturated fatty acids; PUFA, polyunsaturated fatty acids. Image for microniome obtained from Biorender.com. See Discussion for details. **B.** Line graphs showing normalized read counts of select intestinal transporters in various parts of the intestines on the indicated diets (VIV, CO, SO+CO, PL+CO). Line, mean of biological replicates. Genes with significantly different levels of expression between the diets within a given tissue (p-adj ≤ 0.02) are indicated as follows: (VIV, CO, SO+CO, PL+CO). a (VIV vs Co); b (VIV vs SO+CO); c (VIV vs PL+CO); d (CO vs SO+CO); e (CO vs PL+CO); f (SO+CO vs PL+CO). **C.** As in B but for VIV, SO+CO and PL+CO diets. P-adj between SO+CO and PL+CO diets is indicated.

The majority of dysregulated genes can be grouped into one of two categories – metabolism (generally increased) and the immune system (typically decreased) – and are associated with various pathological conditions and diseases ranging from colon cancer, inflammation and IBD to leaky gut and infectious diseases including COVID-19. There were also several genes involved in the metabolism or transport of neurotransmitters – including endocannabinoids, dopamine and serotonin, gamma-aminobutyric acid (GABA), glutamate and glycine – that are dysregulated by the HFDs and could impact brain health. Lastly, we observed changes in a number of transcriptional regulators – including NRs, IRFs, STATs and SREBP1 – that could play a role in regulating the expression of the genes in the other categories (Fig. 8A).

### HFDs impact expression of intestinal genes involved in fatty acid and drug metabolism

Perhaps the best example of a gene involved in fatty acid metabolism that is impacted by diet is *Scd1* which converts saturated fatty acids to monounsaturated fatty acids; it is upregulated by CO more than 10-fold in the jejunum (Fig. 3). Other genes include those that impact linoleic acid and its downstream metabolite arachidonic acid which is associated with pro-inflammatory processes (e.g., *Cyp2c, Cyp2j, Cyp4a, Ephx2*) (Fig. 5). *Ephx2* converts linoleic and arachidonic acid epoxides into bioactive oxylipins; we recently showed that a diet high in SO leads to increased levels of these oxylipins in the intestines and correlates with barrier dysfunction and susceptibility to colitis in mice ^4^. Changes in genes involved in amino acid metabolism (Fig. 5) were less anticipated given that the diets all contained the same amount of protein but an intriguing finding nonetheless as they could play a role in select signaling pathways as noted below.

Dysregulation of numerous genes involved in xenobiotic and drug metabolism – *Cyp, Gst, Ugt* – is consistent with the notion that diet impacts Phase I and Phase II reactions in the liver ^48^ (Supplementary Figures S2,3,4). While we previously reported effects of the CO and SO diets on *Cyp* gene expression in the liver ^2^ and others have reported varying findings in terms of which *Cyp* genes are expressed where in the intestines ^49–52^, to our knowledge this is the first report of different cooking oils impacting *Cyp, Gst* and *Ugt* gene expression in different parts of the intestines. *Cyp2d26*, for example, is upregulated by more than one HFD: its ortholog in humans, *CYP2D6*, is known to metabolize numerous drugs including antidepressants, antipsychotics, analgesics, antitussives, beta-adrenergic blocking agents, antiarrhythmics, and antiemetics ^25,53^ (Fig. 1). These results suggest that the intestines may play a more significant role in drug metabolism than previously recognized and that there could be important health consequences if a basic component of one’s diet - such as cooking oil – changes.

### HFDs impact expression of intestinal genes involved in the immune system, the microbiome and neurological signaling

Given that the intestine is the first line of defense against many foreign invaders and plays a critical role in immune function, it is notable that we identified many genes linked to the immune system that were downregulated by one or more HFD. For example, we observed dysregulation of genes involved in innate immunity (e.g., *Retnlb, Reg3b*), cytokine signaling (e.g., *Ccl8, Ccl20, Ccl22, Tnfsf10*), and pattern recognition (e.g., *Tlr2, Tlr3*) in response to the different HFDs, even without exposure to an external pathogenic agent. *Retnlb* and *Reg3b* both have antibacterial properties and *Reg3b* is regulated by *Retnlb* ^33,54–56^ (Figs. 4–6).

We also found that the HFDs altered the gut microbiome (Fig. 6). While the effects of diet on the microbiome are well established, especially in terms of fiber and polyphenols ^57,58^, less well studied are the effects of different dietary fatty acids. For example, we observed an increase in *Enterobacteriaceae* in the small intestine, a group of organisms known to enhance the inflammatory response ^59^. Further investigation is required to determine whether changes in the microbiome are a direct result of the diets or, alternatively, are a result of changes in the host immune system by the diet which subsequently contributes to dysbiosis. We reported recently that fatty acids such as linoleic acid can contribute to the growth of certain pathogenic bacteria *in vitro*, analogous to changes observed *in vivo* on an SO diet high in linoleic acid ^4^.

Host genes that are implicated in the tryptophan-serotonin pathway, which is known to be impacted by the gut microbiota, were also dysregulated by the HFDs. For example, *Ido1* encodes a key tryptophan-metabolizing enzyme that generates the neurotransmitter serotonin and was downregulated by the HFDs (Fig. 4). In contrast, several neurotransmitter transporters were upregulated by one or more HFD compared to the VIV chow – glutamate transporter *Slc1a3*, dopamine transporter *Slc6a3*, serotonin transporter *Slc6a4* (Fig. 8B). *Faah*, which was greatly upregulated by all three HFDs (Fig. 4), is a hydrolase for endocannabinoids and N-acylethanolamines such as 2-arachidonoylglycerol (2-AG), *N*-arachidonoylethanolamine (AEA) ^60^. This suggests that the HFDs might result in decreased levels of endocannabinoids in the gut which is what we observed with a soybean oil diet ^4^. Although only a total of 55 genes were dysregulated between the SO + CO and PL + CO diets (DUO: 47 genes; JEJ: 2 genes; TI: 5 genes; PC: 1 gene), several of the high LA soybean oil-specific genes were involved in neurotransmitter signaling – glycine transporter *Slc6a9*, GABA receptor *Gabra4*, and *Gatm*, an amidinotransferase involved in creatine biosynthesis critical for cognition, language and behavior. All were significantly downregulated in SO + CO compared to low LA/high oleic acid diet (PL + CO) (Supplementary Tables S2-S5, Fig. 8C). Taken together, the findings from the gene expression and microbiome analyses are consistent with the notion that the gut-microbiome-brain axis may be influenced by what we eat and affect our brain health ^57^. Indeed, we have previously reported that the same diets as used in this study impact the transcriptome of the hypothalamus and many of those genes are related to mental health ^61^.

### HFDs impact expression of genes involved in transcription regulation

One potential mechanism by which different dietary fats could alter the expression of so many genes in the intestines is via nuclear receptors (NRs) which respond to hydrophobic ligands, including fatty acids. While assessing the impact of the dietary fats on the transcriptional activity of NRs in the gut is beyond the scope of this study, we did observe changes in expression of two NRs that bind fatty acids – PPARa and HNF4a – as well as NR co-regulators such as SHP (*Nr0b2*) and PGC1A (*Ppargc1a*) (Fig. 2). HNF4a, down regulated by PL + CO in the duodenum, binds LA and plays a critical role in maintaining intestinal health, intestinal epithelial differentiation and barrier function ^62–64^; it is also dysregulated in colon cancer as well as colitis and is an IBD susceptibility gene ^4,65,66^. Interestingly, *Retnlb*, a known HNF4a target gene ^62^, is also downregulated by the HFDs in the duodenum. *PPARa* binds a variety of fatty acids ^67,68^ and is involved in lipid metabolism as well as nutrient transport and energy; it also plays a protective role against colon cancer ^69,70^ Ppara was upregulated by all three HFDs in the small intestines, as was Cd36, a fatty acid transporter and target of PPARa ^71^ The other most prominent transcription factor family that was dysregulated by the HFDs were the STAT/IRF factors involved in interferon signaling (*Stat1, Stat3, Irf1, Irf5, Irf8*) and hence play a critical role in the immune system. Lastly, SREBPF1, which regulates the expression of fatty acid and cholesterol metabolism genes, including *Scd*1, is upregulated by the HFDs and is potentially linked to COVID-19 (Fig. 7) ^72,73^.

### Impact of HFDs on genes involved in barrier function, IBD and colon cancer

Several diseases, including IBD, colon cancer, and a leaky gut (barrier dysfunction) have been linked to consumption of HFDs and rates of these diseases have been increasing along with increasing fat intake ^9,74^. We observed changes in expression of many genes by one or more HFDs which could contribute to intestinal disease (Figs. 3, 4). For example, there was a decrease in expression of a number of anti-cancer genes including pro-apoptotic gene *Casp3*, DNA repair genes *Mgmt* and *Parp1* and tyrosine phosphatase *Ptpn11*. There was also an increase in expression of several cancer-promoting genes such as intestinal stem cell marker *Lgr5* and *Vnn1* (vanin1, a biotinidase). In other cases, the HFDs seemed to be protective. Decreased expression of *Duox2* in HFDs suggests a lower inflammatory response compared to the control diet ^75^, which could be beneficial in halting the progression of colorectal cancer ^76^, and reduced expression of *Ripk3* (receptor-interacting protein (RIP) family of serine/threonine protein kinases) in the terminal ileum may help alleviate inflammation in IBD ^77^. Some genes showed differential effects depending on the HFD. For example, both *Cldn10* and *EGF* have lower expression in SO + CO vs PL + CO: reductions in both of these genes can impair barrier function. This is consistent with previous findings from our lab and others that a high LA diet can contribute to barrier dysfunction while olive oil, a key feature of the Mediterranean diet, is considered to be anti-inflammatory ^4,78,79^.

### Effects of HFDs on COVID-19-related genes

HFDs, including the ones analyzed in this study, often contribute to obesity which is a significant risk factor for COVID-19 ^80^. COVID-19 patients can experience gastrointestinal symptoms, including damage to the intestinal epithelial barrier ^81^. Therefore, it is perhaps not surprising that the lower gastrointestinal tract has a large number of ACE2 receptors and that its expression, along with the genes that encode accessory proteins ENPEP and SLC6A19 (BOAT1) which facilitate viral entry via ACE2 ^82,83^, is increased in one or more of the HFDs (Fig. 7). Like ACE2, *Klk1* and *Klk1b5* are part of the RAS pathway and are thought to be required for viral processing ^84^; their expression was also increased in the CO diet. Furthermore, several host genes involved in the immune response to SARS-CoV-2 are downregulated by one or more HFD – *Gzma, Irf1, Stat1, Stat3*. In addition to dysregulated gene expression, several of these COVID-19-related proteins have also been found to interact with one or more SARS-CoV-2 viral proteins. Taken together, our results suggest that these HFDs, or the metabolic dysfunction and/or the dysbiosis caused by them, might be detrimental to COVID-19 patients ^85,86^. While additional studies are required, it is nonetheless intriguing to speculate that effects of a high fat diet on the intestinal tract could potentially account for some of the demographics of the pandemic across different populations ^87,88^

### Limitations and caveats

Limitations and caveats to this study include the length of time on the diets (24 weeks) – the observed changes in gene expression could be due directly to the diets and/or to their long-term effects such as obesity, diabetes and susceptibility to colitis ^2–4^. Others have shown changes in gene expression and the microbiome after just a few days on a HFD, which could be a reflection of the body adjusting to a new nutrient environment ^12^; the effects we observe after 24 weeks may represent more persistent effects on gene expression. In addition to increased fat content, the HFDs were also different from the low-fat Viv chow control in that they did not contain fiber. That being said, we have shown that an SO-enriched diet containing fiber leads to similar increases in susceptibility to colitis in mice and causes a similar amount of weight gain as one without the added fiber ^3,4^. Additionally, even though all three HFDs lacked fiber, they often displayed different effects on gene expression suggesting that not all of the effects observed are due to a lack of fiber. This study examines RNA levels only, which may not always relate to protein levels – e.g., *Cyp2c55* has very high levels of RNA in the proximal colon but its protein levels are reported to be very low, similar to the small intestines ^49,50^. Whole tissue was used, so in addition to intestinal epithelial cells, other cell types including immune cells would have been sampled. Single-cell RNA-seq by others show that a HFD does indeed impact different cell types in a differential fashion, and differences can be observed within days ^12^. Finally, the relevance to humans must be established. Since most of the DEGs highlighted in the study are highly conserved between mouse and human, including several of the transcriptional regulators – HNF4a, PPARa, STAT1/3, IRF1, SREBPF1 are all over 80% identical between human and mouse on the protein level– we anticipate that many of the effects reported here will also be found in humans.

## Data Availability

All data generated or analyzed during this study are included in this article and its Supplementary Information Files. The raw data RNA-seq data are publicly available in Gene Expression Omnibus (GEO), accession number GSE220302. DNA sequencing data of the microbiome is publicly available at SRA BioProject, Accession #PRJNA615924.
